# The Role of Patient Organizations in Shaping Research, Health Policies, and Health Services for Rare Genetic Diseases: The Dutch Experience

**DOI:** 10.3390/genes15091162

**Published:** 2024-09-03

**Authors:** Ysbrand Poortman, Martina Ens-Dokkum, Irmgard Nippert

**Affiliations:** 1 VSOP, 3528 BO Utrecht, The Netherlands; 2 Kentalis International Foundation, 2716 NR Zoetermeer, The Netherlands; ensdokkum@gmail.com; 3 Curium-Leiden University Medical Center, 2342 AK Oegstgeest, The Netherlands; 4 Faculty of Medicine, University of Münster, 48149 Münster, Germany

**Keywords:** patient organizations for rare genetic diseases, diagnostic odyssey, multi-stakeholder cooperation, genetics and genomics health policies, genetics and genomics health services, civil society engagement

## Abstract

In 2023, the genetics scientific community celebrated two special anniversaries: the discovery of the double helix structure of DNA was published in 1953 and in 2003 the Human Genome Project was declared completed and made publicly available. To this day, genetics and genomics research is continuing to evolve at high pace and is identifying a steadily increasing number of genes as causal for distinct genetic diseases. The success story of genetics and genomics would not be complete without taking due account of the role of patient advocacy organizations in this process. This paper is based on the personal narrative (oral history) of a father whose daughter was born with a rare genetic disease (RGD) in the 1960s. The first-hand experience of living as a family with an RGD in those days made him a leading pioneer not only in the foundation of patient organizations at national, pan-European, and international levels but also in the development of multi-stakeholder co-operation and networking. Today, patient advocacy organizations play an active role in shaping health and research policies at national, EU, and international levels to ensure that their needs in regard to advancing RGD diagnostics, care, and treatment are addressed.

## 1. Introduction

In 2023, the genetics scientific community celebrated two special anniversaries. The discovery of the double helix structure of DNA was published in 1953 as a one-page article in Nature [1]. In 2003, the Human Genome Project, generating the first sequence of the human genome, was declared completed and made publicly available [2].

To have gone from the discovery of the double helix structure of DNA to the assembly of the human genome’s roughly three billion nucleotides in fifty years stands for stunning scientific developments. Both landmark events initiated and accelerated a string of major scientific achievements leading to impressive advances in genetics and genomics science, supported by tremendous progress in genomic technologies. Genomics started as a journal title, coined by Tom Roderick in a Bethesda saloon in 1986, and soon became a whole new science discipline [3,4].

Thanks to the completion of the draft human genome sequence, many of the obstacles to disease gene mapping were reduced. High-throughput sequencing technologies and the availability of reference genomes from diverse populations have led to an estimated fourfold increase in the discovery of rare disease-causing genes between 1999 and 2019, allowing earlier and faster diagnosis for more genetic diseases in clinical care [5]. To this day, genetics and genomics research is continuing to evolve at high pace. This dynamic is turbocharging the ability to decode DNA and is keeping the number of genes identified as causal for distinct genetic diseases steadily rising [6,7].

In 2020, Nguengang Wakap et al., published data on 6172 distinct rare diseases (RDs) contained in the Orphanet database, 71.9% of which were distinctly genetic (RGD) and 69.9% of which were exclusively paediatric onset. The study estimated that RDs affect at least 3.5–5.9% of the worldwide population and that this point prevalence translated into “conservative” figures of 18–30 million persons in the European Union (EU)/Europe, and 263–446 million people affected worldwide [8].

Against this historic background, this paper discusses the parallel evolvement of RGD patient organizations. The stunning success story of genetics and genomics during the last seven decades would not be complete without taking due account of the effective role of patient advocacy organizations in shaping genetics and genomics research, health services, and health policies. In 1953, RGD patient organizations were still few and far between. In the United States, the Muscular Dystrophy Association was founded in 1950 and the Cystic Fibrosis Foundation was established in 1955 [9,10]. In Europe, the first RGD patient organizations were founded in the late 1950s and 1960s [11]. Patient organizations gained traction in the 1970s and 1990s. Since then, exponential growth in the number of patient groups has been observed and, as of today, numbers are still on a continually upward trajectory. Ultimately, the dynamic “growth of patient organizations is a testament to the dynamic scientific progress in genetics and genomics over the past decades and its growth signifies the vitality of civil society, driven by families and patients affected by rare diseases” [12].

### Aims and Objectives

The overall aims of this paper are: (i) to inform the genetics and genomics scientific community about the historic development and growth of RGD patient organizations since the discovery of the structure of DNA; (ii) to describe the evolution of increasingly empowered RGD patient organizations at national, pan-European, and international levels, and their impact on health services and health policy development; (iii) to address the rise of needs-based and patient-led research and the engagement in multi-stakeholder networking and co-operation to advance diagnostics, care, and treatment for RGDs; and, overall, (iv) to demonstrate that RGD patient advocacy organizations and their members have successfully achieved a paradigm shift in the role of patients both by acting as de facto experts on their respective diseases and by effectively promoting their research and health policy agendas.

## 2. Materials and Methods

### 2.1. Key Methods

This paper is based on the personal narrative (oral history) of a father whose daughter was born with an RGD in the 1960s. His first-hand experiential knowledge of living as a family with an RGD in those days made him a leading pioneer not only in the foundation of patient organizations at single RGD, national, pan-European, and international levels but also in the development of strategies for and models of multi-stakeholder co-operation. He served as an advisor to his national government, was appointed by Royal Decree a member of the Health Council of The Netherlands (2000–2008), and closely collaborated with the genetics scientific community on the creation and implementation of genetic services at university centre/academic hospital levels. For contributions of national significance for The Netherlands, he was appointed an officer in the Order of Orange Nassau.

His story serves as “*pars pro toto*” for the empowerment and engagement process of RGD patient organizations that has taken place during the last 60+ years, i.e., since the time his daughter was born. The story is told from the perspective of a contemporary witness. The concept and content of the paper were discussed during several online and face-to-face meetings with external advisors. However, the final content and design of the paper lie solely in the responsibility of the corresponding author.

### 2.2. Material

The paper relies on historical sources including (i) the corresponding author’s personal papers and documents, (ii) the archives of the organization for muscular diseases (V)SN (Spierziekten Nederland), (iii) the archives of the VSOP, the Dutch national alliance for rare and genetic diseases, including a report published on occasion of the VSOP’s 25th anniversary [13], (iv) national government advisory papers and EU (European Commission) reports and policy statements, and (v) other (scientific) publications. For information on recent and ongoing developments within (V)SN and VSOP, their current managing directors were consulted.

## 3. Results

### 3.1. The Beginning—A Father’s Story

Our first child, a daughter, was born healthy in September 1963. She was a happy, vivid baby and developed normally in every area during the first nine months of life, even crawling up the stairs. From that time on, we increasingly got a feeling something was not right: her legs seemed to collapse during walking and her motor development slowed down. As our anxiety grew, we decided to take her to the GP shortly after her first birthday. He told us not to worry: “Some children simply develop not as smoothly as others”. However, our anxiety continued to increase in the following months because her motor development declined, while her language and speech development appeared remarkably good, and we insisted on referral to an expert. She was admitted to the neurological department of a general hospital, where she was placed among adults. Visiting her after two days, we discovered that she was tied to the railing of her bed out of fear she would climb over it and fall. We complained to the nurse, telling her she was unable even to stand up. Asking for information from the neurologist, we were told he was on holiday for a month. She was released from her ties but had to stay in hospital, although diagnostic procedures only started after that month.

That being achieved, in May 1965, the neurologist told us, “It is a nasty disease, we are not able to help you, and I am not going to mention the name of her disease because then you would go looking for information in medical literature, and that would not bring you any good”. Because we continued to insist on a clear diagnosis, he referred us to a well-known neurologist at the Academic Hospital in Amsterdam. After checking the histological sections he had received from her neurologist and the pathology information, he reported to our great distress that it was Werdnig–Hoffmann syndrome and told us, “It is a lethal muscular disease, nothing to do about it but pray, with only two more cases known in The Netherlands”. A follow-up appointment would serve no use, in his opinion: “She is going to die anyhow”.

I refused to accept this verdict and went on searching for a more satisfying answer to my questions about diagnosis, prognosis, and treatment options. Therefore, I travelled around Europe during our holidays with my wife and daughter to visit all kinds of ‘experts’, recognized as well as self-professed, regular, alternative, clairvoyants, and so on. We managed to find and visit well-known medical experts. In the summer of 1965, we first went to the annual meeting of the Belgian muscle diseases association, the existence of which I had learned during my asking around. My intention to speak to the professor who chaired it failed because he left the meeting in a hurry, but on the other hand, we won the first prize of a lottery held there: a living sheep! To our daughter’s regret, we could not take it home and had to give it back to the association. From there we went to Professor Thieffry in France, who was known as a specialist on neuromuscular diseases. He gave us another name for her disease, Wohlfart–Kugelberg–Welander (WKW) syndrome [14], but not before using her as a demonstration model patient for his students, which she did not mind at all, by the way. We were so excited because he told us it was not lethal that we forgot to ask for more information. Back in The Netherlands, we first returned to the general neurologist who had examined her before and told him the name for her disease we had just heard. He responded with “Never heard of it”. Then we went to the neurologist in the Academic Hospital. His verdict was, “That’s not it”.

Hoping to sort out which opinion was the right one, we visited yet another expert, Professor Walton in Newcastle, UK, who had written two handbooks about neurological diseases, after having received his written consent to visit him in spring 1966. He confirmed the diagnosis of WKW syndrome, in our opinion a satisfying diagnosis at least with respect to survival. Disturbingly, however, looking at my wife, who was visibly pregnant at that time, he told us WKW was a genetic disease. He advised us to look for parents of children with the same or a similar disease, and even put us in touch with such a family.

We then realized all the more that we knew far too little about significant aspects of the disease and decided we had to look for those experts after whom the disease was named. We managed to find their addresses via the Swedish embassy. Kugelberg was excluded for our purpose because it turned out he was an electrophysiologist. Professor Wohlfart lived in Lund, and Professor Welander in Umea, that is almost in Lapland. Later on that year, we went on our way to Scandinavia in our mini-caravan with our daughter, her baby brother, and a babysitter. Although we got stuck in the snow 40 km from Copenhagen, we were able to make it to Lund. There it appeared that Professor Wohlfart had passed away the week before our arrival. Doctor Gamstorp, second in command in the paediatric department, had taken over. She confirmed the diagnosis and prognosis, told us that the risk that our next child would have the disease was 25%, and that much was still under investigation. We were instructed to contact a paediatrician, a physiotherapist, and a genetic counsellor when back in The Netherlands.

### 3.2. Challenges of Living with a Rare Genetic Disease: Unmet Needs and Founding a Parent/Patient Organization for Neuromuscular Diseases

We were not the only ones who had to find our way to diagnosis, prognosis, and care alone. In 1967, a mother of a child with muscular dystrophy was interviewed on Dutch television. She addressed her difficulties and problems: loneliness, lack of support, and feelings of being left out. I responded to her (as did many other parents). We shared the same experiences and faced similar challenges: going through a lengthy difficult diagnostic odyssey that lasted for years before obtaining a correct diagnosis, and encountering a plethora of medical knowledge deficiencies resulting in misdiagnosis and leading to a delayed correct diagnosis. In addition, we were facing a persistent lack of appropriate, reliable, and actionable information on issues relevant to us and our families such as prognosis, the spectrum of the disease, heredity, treatment options, the risks of primary and secondary disability and possibilities to prevent or ameliorate these, and (para)medical and psycho-social support.

When we recognized the commonality of our experiences, we decided to break individual isolation and to address unmet needs and concerns collectively. We recognized the pressing need for a knowledge and psychosocial support centre for families living with neuromuscular diseases, and decided to take action. In the same year (1967), we organized a meeting with around a hundred participating parents and patients, among them a representative of Muscular Dystrophy UK [15]. This was the start of the parent/patient organization for muscular diseases in The Netherlands, Vereeniging Spierziekten Nederland, or (V)SN, in which I, being a full-time teacher in biology at that time, took a leading position, focusing on disease-specific medical and international issues, while other members concentrated on psycho-social and care issues [16]. Our mission statement was as follows: “To promote the interests of patients and carers, and to treat and prevent the disease”.

Meanwhile, my wife and I faced more challenges to cope with in our daily lives as a family. Our daughter always kept her good moods, but we literally had to carry her growing burden because she could not use her muscles, causing a lot of strain to our backs. We realized we lacked advice on how to lift and carry her in a way that did not ruin our backs. Daily care for her and visits to (para)medics took a lot of our time. We regularly had to ask for time off from work. Around three times a week, our daughter had to visit the revalidation centre for physiotherapy, for prescriptions, and for orders for corsets and wheelchairs. The delivery of such essential devices often took such a long time that she had almost or completely outgrown them by the time they finally arrived. In between, we took her to her regular primary school, which was relatively easy for us because we both were teachers and worked in that building. This made it easier for us as well to discuss accessibility of the building and toilet for her wheelchair, and to help when she had to use the toilet. We bought her a recorder and arranged for lessons as the physiotherapist told us this was a playful way to develop her lung volume. As we were told as well that swimming would make it easier for her to move, we built a swimming pool within the house. Since the health insurance did not pay for most extras she needed due to her disease, I had to pursue many legal actions.

The experience of juggling care and daily life helped me to translate the fairly general mission of (V)SN into a more detailed strategic long-term action plan defining (V)SN’s major goals and objectives. The overall plan was complemented by strategic operational steps required to achieve our main goals and objectives, namely to:

1. Address the knowledge gap and the lack of informed care; provide appropriate, actionable education and information for parents about home care, transportation, technical help, financial help, and recreation; and organize support by setting up mutual help groups for different neuromuscular diseases;
2. Promote needs-led research, cooperation, and active involvement in the field of research and in the development of therapies;
3. Generate attention and publicity to reach all involved patients and parents and to find financial resources to support (V)SN’s activities.

As there were no examples or models of other patient organizations around to take lead or inspiration from, the experience-based action plan became, so to speak, a kind of blueprint for the further goal-oriented strategic development of (V)SN.

#### 3.2.1. (V)SN: Early Milestones and Achievements

In 1972, (V)SN’s membership had increased to 550. Finding a balance between peer-to-peer support and upskilling members, between focussing on genetic muscular diseases alone and on a wider spectrum of muscular diseases, became a challenge and needed to be addressed. To achieve all goals and objectives, despite growing membership and lack of time of board members, we took further steps in strengthening (V)SN’s infrastructure and capacity building by:

- Implementing standing working groups to realize home visits for advice by an experienced member plus supplementing telephone consultation to fill home visit gaps;
- Organization of a special symposium aiming at the creation of a professional structural framework for (V)SN;
- Establishing a committee to raise attention for genetic muscular diseases in the medical and scientific communities;
- Organizing regular meetings with medical experts and researchers to facilitate members’ access to state-of-the-art information, to keep members informed and updated on current research projects and on latest research findings, and, last but not least, mentoring medical experts on patients’ needs and advocating needs-led research.

By developing these capacities, we were able to start a multi-disciplinary diagnostic working group, enabling us to attract more attention from academic hospitals for RGDs. For parents, we offered regular advice and training by physiotherapists for prevention and treatment of secondary disabilities (contractures). We gained more publicity and increased public awareness through our brochure *Early Detection of Genetic and Inborn Diseases*, leading to an article in a national newspaper with the heading: “Parents inform physicians”. (V)SN appeared to meet the information needs of doctors, paramedics, and other healthcare providers. It got three reprints [13].

We collaborated with geneticists in outlining a plan for clinical reference centres for specific RGDs at the eight university hospitals in The Netherlands. From 1991 onwards, the plan was implemented and laid the foundation for the research and care infrastructure for patients with muscular diseases in The Netherlands. The infrastructure is characterized by a division with regard to focus on specific RGDs among universities, which still operates today.

As of today, there are six certified centres of expertise (CoE) for neuromuscular diseases (NMD) across The Netherlands [17]. Together, they are a partner within the Dutch Neuromuscular Centre [18]. The legal national basis for the accreditation of a CoE in the EU is based upon the 2009 European Council recommendation on an action in the field of rare diseases [19,20].

Since 2017, the Dutch Neuromuscular Centre has been a member of the European Reference Network (ERN) [21] for Rare Neuromuscular Diseases (ERN-EURO-NMD) [22]. ERNs were launched in 2017. From the very beginning, patient organizations have played an active, instrumental role in their development [23]. EURO-NMD is one of the first and largest ERNs, bringing together 82 healthcare providers across 25 European countries and 27 patient organizations [24]. Today, in The Netherlands, the involvement of patient organizations in connecting a CoE with an ERN is obligatory [25].

#### 3.2.2. (V)SN Involvement in Orphan Drug Development

In 2001, I officially left (V)SN. My successor, Marcel Timmen, followed (V)SN’s path towards screening and drug development for NMDs, which had already started in the late 1990s. (V)SN receives programmatic funding for both basic and clinical research through collaboration with the Princess Beatrix Fund for muscular diseases; this was supplemented by a program for gene-therapy development in 2021 [26]. Funding obtained by the Princess Beatrix Fund led to the relatively early development of an orphan drug for Pompe disease by the Pharming biopharmaceutical company from The Netherlands. The first trial with three infants took place at the Erasmus Medical Centre in Rotterdam in 1999 [27]. The same year, at (V)SN, patients from around the world came together to join forces and to found the International Pompe Association (IPA) [28].

As the drug was produced using genetically modified rabbits (transgenic rabbit milk), the import was not easy: it had to be accorded by the Minister of Agriculture. Action groups opposed the production of a drug through animals. However, due to the lobbying of (V)SN, their actions remained unsuccessful. Today, the Pompe Centrum is the national centre of expertise for Pompe disease. All Pompe disease patients in The Netherlands are referred to this centre [29].

The next problem (V)SN took action on was a negative assessment of the cost-effectiveness of this orphan drug by the board of health insurers of the Health Insurance Fund (College voor Zorgverzekeringen, CVZ, since April 2014 renamed Zorginstituut Nederland). This resulted in their proposal to stop reimbursement for some Pompe patient groups. The CVZ refused to withdraw its proposal, but finally made a U-turn and advised the Minister of Health to maintain the reimbursement of the therapies for all patients [30].

Today, 180 different drugs and therapies are in the pipeline for 20 different muscle diseases. (V)SN is committed to ensure their rapid availability through:

- Contacting and lobbying manufacturers to prepare for their assessment of cost-effectiveness and reimbursement procedures in The Netherlands;
- Lobbying the National Health Care Institute (Zorginstituut Nederland) [31] and the Ministry of Health (VWS) of The Netherlands [32];
- Educating physicians and researchers with respect to their role in the admission and reimbursement of orphan drugs. (V)SN is committed as well to the management of expectations of patients with regard to gene therapy.

#### 3.2.3. (V)SN: Newborn Screening for Spinal Muscular Atrophy (SMA)

Three SMA treatments, albeit with different modes of application, to increase the availability of functional SMN protein, have received market authorization for use in the European Union (EU) from the European Medical Agency (EMA): nusinersen (Spinraza^®^), risdiplam (Evrysdi^®^), and (Zolgensma^®^).

In 2019, the national Health Council of The Netherlands positively advised the inclusion of SMA screening in the Dutch newborn screening program [33]. Through lobbying by (V)SN, the heel-prick screening trajectory process gained momentum in The Netherlands when Zolgensma^®^, the first approved gene therapy for a neuromuscular disease, entered European markets in 2020. 

Zolgensma^®^ gene therapy is a one-time therapy that targets the cause of the disease and seems to prevent it [34]. It is an option for babies with presymptomatic 5q SMA with a bi-allelic mutation in the SMN1gene and up to three copies of the SMN2gene. Newborns with SMA can now—newborn screening for SMA has been available in The Netherlands since July 2022 (neonatal bloodspot screening program)—be identified within a few days of birth and referred to the SMA expertise centre for just-in-time treatment/gene therapy with Zolgensma^®^. A short trajectory is imperative as this gene therapy is most effective when there is still no or very little irreparable nerve damage [35,36].

However, Zolgensma^®^ is one of the world’s most expensive drugs. The drug costs approximately €1.9 million per course of treatment [37]. In 2021, the Beneluxa Initiative, a multi-country negotiation platform jointly created by Belgium, Ireland, and The Netherlands, reached a willingness-to-pay agreement on the pricing of Zolgensma^®^ [38,39,40]. Zolgensma^®^ is now reimbursed for two specific groups of young patients in all three countries [35,41].

The following bottlenecks regarding the availability of SMA treatments requiring attention from (V)SN have been identified: Their high costs and limited available evidence from manufacturer studies, especially evidence gaps concerning safety and efficacy. Many uncertainties are due to the short-term studies of manufacturers. Available clinical trials cover only a subgroup of patients, mostly in the early stages of the disease and with a short observation period [36]. Another current bottleneck is the strict assessment of eligibility for treatment by the National Health Care Institute of The Netherlands in response to the “established state of medical science and medical practice” [35].

The consequences of these bottlenecks are shown in the treatment algorithm of SMA. Each of the three drugs now available for SMA in Europe is only available for a small, defined group of patients, leading to a highly fragmented treatment algorithm and to a group of around 20 patients who cannot claim any treatment based on the current reimbursement schemes. This has triggered discussions on the appropriate selection of patients for treatment in the context of limited available evidence. Arguably, industry, national health-care systems, and patient organizations will all play a crucial role in redefining the role of current and new disease-modifying therapies in the near future [35,36].

#### 3.2.4. (V)SN Today

(V)SN now has approximately 9000 members [42]. More than 300 volunteers actively support the association’s activities. (V)SN maintains a network of 10 regional groups and 15 national, diagnosis-bound support groups. These groups monitor international developments in the field of research, are involved in psychosocial care, and provide information on technical appliances [43]. Stimulating continuous scientific research has been one of the most prominent objectives of (V)SN since its foundation in the 1960s [44].

At European level, (V)SN represents Dutch patients within the European Reference Network for Rare Neuromuscular Diseases (ERN EURO-NMD) to ensure that the needs of people living with a rare neuromuscular disease are included in the strategic and operational delivery of the network [42].

#### 3.2.5. Neuromuscular Diseases Internationally United, Networking and Partnering—From a Patients’ Initiative to Pan-European Research Institutions: Establishing the European Alliance of Neuromuscular Disorders Associations (EAMDA) and the European Neuromuscular Centre (ENMC)

After establishing (V)SN’s national infrastructure for patient involvement and for national multi-stakeholder collaboration, initiating international multinational collaborative networks of NMD stakeholders as second-level European organizations was just the next logical step. (V)SN took the lead in founding the European Alliance of Neuromuscular Disorders Associations (EAMDA) in 1971 in London [45]. The purpose and design of EAMDA is based on a partnership model [46]. EAMDA’s overall objective is to ensure multi-stakeholder collaboration by bringing together patients, families, healthcare professionals, researchers, and other stakeholders to share information, discuss the latest research findings, and promote the best practices for the diagnosis, treatment, and care of individuals with NMDs.

In 1992 we set up the European Neuromuscular Centre (ENMC) to stimulate international research, notably through the organization of scientific workshops, an information gateway, and a clinical trial network [47]. To date, nearly 2000 scientists have taken part in more than 270 international scientific consensus workshops on diagnostic criteria, clinical trials, and disease gene identification. A 25-year evaluation revealed that publications derived from ENMC workshops scored ‘high impact’ as illustrated by a mean normalized citation score of 1.24. Also, 16% of the ENMC papers belong to the top 10% most cited articles in the neuromuscular field. The main outcome of the personalized survey was that 90% of all workshop deliverables were started and either ongoing or completed. Of these deliverables, 78% were implemented in the field, bringing state-of-the-art knowledge and new collaborations to researchers and clinicians, improving the designs of clinical trials, and innovating tools to make accurate diagnoses. Workshop reports are amongst the most cited articles in the Neuromuscular Disorders journal [48,49,50,51,52,53,54].

Today, diagnostic working groups from (V)SN have connected and are united in international contexts with 12 European umbrella patient organizations for muscle diseases, such as SMA Europe [55] and Euro Ataxia [56], with the aim to solve bottlenecks in the development of orphan drugs and to ask for their availability in all European countries. Funding and generating patient-relevant research, improving treatment and care, and learning from each other’s expertise are high on their agenda as well.

### 3.3. Founding the First National Patient Umbrella Organization for RGDs in Europe and Shaping the Infrastructure for Genetic Services and Counseling Centres in The Netherlands

By 1975, several other specific-RGD patient organizations had developed in The Netherlands. Our shared interest in exerting and increasing our influence on national policy developments and having an impact on national political decision-making led, after four years of careful deliberations, to the official founding of the national Dutch umbrella organization for rare and genetic disorders, VSOP, in 1979 (Dutch abbreviation of Vereniging Samenwerkende Ouder- en Patiëntenorganisaties; in English, Association of Co-operating Parent and Patient Organizations). It was the first of its kind in Europe, serving to provide patients and caregivers with a common voice and to gain visibility, and I became the managing director [13,57,58].

#### 3.3.1. Shaping and Implementing Genetic Service Centres, Including Genetic Counselling Centres in The Netherlands

At the beginning of the 1970s, it became obvious—at least for researchers working in basic genetics research at university level—that the future demand for genetic services in medicine would increase substantially for both laboratory services (e.g., chromosome analyzes) and for counselling services after diagnostic outcomes. Hans Galjaard, at that time professor at the Erasmus University Rotterdam, realized “that basic research in genetics would be endangered if it took too many of our people to do genetic services” [59].

According to his narrative, he addressed his prediction and concerns in a letter to the Ministry of Health, but did not get an answer from the Ministry, not even an acknowledgement that his letter was received. He talked about this to the then-chairman of the Health Council, a professor of internal medicine in Leiden named Haex. Haex listened and went to the top of the Ministry of Health and invited them to his office in Leiden, and invited Hans Galjaard as well. The joint meeting resulted in an official advisory committee formed in 1973 tasked to prepare an outline of the needs of comprehensive clinical genetics centres, including counselling services, affiliated to all (then eight) University Medical Centres across The Netherlands. Hans Galjaard became secretary of that committee. As a next step, he contacted experts in cytogenetics and biochemical diagnostics and various clinicians at the different medical academic centres to prepare an outline of their needs. It took the committee four years to prepare the *Advies inzake Genetic Counseling* report for the Health Council (Gesondheidsraad) [60]. One of Galjaard’s remarkable achievements was that, from the beginning, he closely worked together not only with his peers but also—from the very start—contacted me to discuss patient advocacy, strategically connecting clinical expertise with patient expertise.

One of Hans Galjaard’s other notable achievements was to work closely with the health insurance system leaders to convince them of the benefits of medical genetic services and to encourage them to fund its development and services at all medical academic centres across The Netherlands. This collaboration started even before the advisory report to the Health Council was finalized. When the report was ready, he became the only negotiator for funding the whole of genetics services in The Netherlands. The negotiations took about two years. In total, these steps resulted in a carefully planned system of sharing responsibility for different major disorders, linked to research in the particular field and ensuring that high-quality services and research were spread across the country and wasteful duplication avoided. I then started—partnering with Hans Galjaard—to set up the centres for genetic counselling at all national academic hospitals, with patient representation on the boards of all of these. Instead of ‘advice on heredity’, which we considered too directive, we propagated ‘education on heredity’ (Dutch: ‘Erfelijkheidsvoorlichting’) [13]. Multiple appearances of both of us, Hans Galjaard and me, in discussions and articles in the media created nationwide public attention to the role of genetics in health and for RGDs. Genetic counsellor training in The Netherlands began in the early 1990s. Early trainees were nurses or other healthcare assistants who received on-the-job training by clinical geneticists [61].

Hans Galjaard’s farsighted planning skills earned him an international reputation. An anecdote that casts some revealing light on the state of genetic service provision in the early 1980s was told by him during an interview with Peter Harper in 2011. In 1983, the United Nations had asked him to go to China to provide advice to the Chinese government on implementing genetic services. In Galjaard’s own words: “*They invited me to advise them. China was quite closed at that time and there were no civil airplanes yet. We flew in military airplanes and people were all dressed in Mao costumes. At the end of the trip, we met the wife of the president of China, who was in charge of Mother and Child Health. She said to me at dinner ‘And how many genetic centres you recommend for our country?’ And I said, Well, in Europe there is a rule made by Passarge [Eberhard Passarge a US-trained German human geneticist] at that time: one per 2 million inhabitants, roughly. In Holland we have 8 centres for 16 million people. So you should have 500 or 550. And then she looked at me and said, ‘Let’s start with 2’*” [62].

#### 3.3.2. Key Steps Taken by VSOP to Promote Genetic Counselling Services and to Raise Public Awareness about Genetics

(i) Raising public awareness, addressing “genetic illiteracy”

From the mid-1980s onward, VSOP took an active role in educating the public about genetics. At that time, genetic illiteracy was widespread in The Netherlands, not only in the public but also in the medical profession, and among political decision-makers in particular. This was explained by Leo Ten Kate, a Dutch geneticist, in terms of a background of fear and unease regarding genetics in Dutch society [63,64,65].

Actions taken by VSOP to increase genetic literacy include the publication of an Ethical Manifesto in 1990 (“Ethisch Manifest van de VSOP”), which was developed by joint VSOP working groups from 1986 to 1990 in response to a governmental report suggesting that genetic services should prevent the birth of newborns with genetic diseases instead of offering information for autonomous informed decision-making [13].

Information provided for teachers for the curriculum of secondary schools started in 1986 [13]. In addition, we published the book *Heredity and Disease*, which not only received much attention in the national media but was also widely used in education about heredity [66]. National media campaigns were started in 1993 and continued until 2000. They were divided into three phases covering the periods before and during pregnancy and on the first years of a child’s life. In the first phase, emphasis was on lifestyle, the possibility of clarifying genetic risks, e.g., carrier status, and making parents aware of their own personal choices in the prevention of congenital disorders with messages like “Genetic counselling, make sure you know the way” and “What you should know about heredity before you consider having children”. In the second phase, prenatal diagnosis was discussed, but also healthy nutrition, the use of medication, working conditions, harmful substances, and other health topics. Sixteen themed pamphlets were made available free of charge for public information through support from the Dutch Prevention Fund. The third phase targeted the medical profession. A congress titled “Between Chances and Risks” focused on early detection of congenital disorders and on referral pathways, correct treatment, and guidance [13].

Since 1993, VSOP has been providing information leaflets on heredity in several languages addressing migrants in The Netherlands [13]. A detailed overview on publications by VSOP to raise public awareness is documented in *Erfelijkheid op de Agenda, Actualiteit van 25 jaar VSOP* [13]. VSOP was actively involved in the founding of the Dutch national genetic resource and information centre, Erfocentrum. Nowadays (since 2000), it is an independent state-funded centre that enjoys great public attention [67].

(ii) Capacity building for genetic services

Between 1987 and 2004, VSOP appointed eleven professors as ‘Bijzondere Leerstoelen’ (professors by special appointment), or ‘VSOP hoogleraaren’, at almost all national universities, including the first female professor in clinical genetics in The Netherlands [13].

#### 3.3.3. National Umbrella Patient Organizations Internationally United and Partnering

Recognizing the EU added value, i.e., that the sum of actions taken together will lead to better overall results than individual groups at single country levels can yield, we decided in the early 1990s that internationally united national umbrella patient organizations for RGDs would strengthen our ability to generate more visibility, attract more attention, and increase our ability to address patients’ unmet needs at health policy levels in the EU, in Europe, and beyond. This holds especially true in regard to the low prevalence of RGDs and the scarcity of health experts for RGDs scattered throughout European countries. As a consequence, just as with (V)SN, I expanded VSOP’s scope internationally and co-founded new European umbrella organizations (third-level organizations) of umbrella patient advocacy organizations. For example:

In 1991, VSOP and other European RGD patient organizations were invited for the first time to the European Society of Human Genetics (ESHG) annual conference, and there, acknowledging increasing integration processes at EU level, a preliminary meeting to consider forming a pan-European patient organization was held. VSOP took the lead. The UK’s Genetic Interest Group (GIG, founded in 1989, since 2010 named Genetic Alliance UK) initially declined the invitation to join but later overturned its decision and became a founding member of the European Alliance of Genetic Support Groups (EAGS) when it was officially established at the next ESHG meeting in Copenhagen in 1992 [68,69].

This was the start of a strong working cooperation between The Netherlands (VSOP) and the UK (GIG/Genetic Alliance UK). Strategically, we wanted to put the unmet needs of the estimated 20–30 million European RGD patient community on the European/EU health policy, health services, and health research agendas. We intended to be included in shaping these agendas and to play an active and instrumental role in the process.

EAGS worked closely together with the ESHG. In 2004, EAGS changed its name to EGAN, the European Genetic Alliances’ Network [70]. EGAN was and still is represented on several committees of EMA, like the Committee on Orphan Medicinal Products (COMP) and the Committee on Advanced Therapies (CAT). In the past, EGAN participated with similar organizations from Ukraine, Africa, the Middle East, Asia, Australia and New Zealand, North America, and South America in the International Genetic Alliance (IGA) [71]. When active in European projects, VSOP acts on behalf of EGAN [72].

In addition to multinational patient organizations, VSOP contributed actively to the founding of several other multi-stakeholder partnerships, namely:

(1) The European Forum for Good Clinical Practice (EFGCP, 1993), aiming at ethically sound clinical research, protecting the rights, safety, and well-being of research participants [73]. The forum provides patients with knowledge and skills to engage in drug research and development and health technology assessment, and educates researchers on how to engage with patients;
(2) The European Platform for Patient Organizations, Science and Industry (EPPOSI, 1994) [74]. EPPOSI is an independent, not-for-profit, partnership-led, and multi-stakeholder think tank based in Brussels, Belgium. EPPOSI’s goal is to develop multi-stakeholder perspectives on biomedical innovations, and it is working at the “cutting edge” of European health policy-making with the overall aim of “bridging the gap between innovation and improved public health outcomes”;
(3) The World Alliance of Organizations for the Prevention and Treatment of Genetic and Congenital Conditions (WAO), which was founded in 1994 on the initiative of March of Dimes (USA) to develop a perspective on genetic innovations from both the academic and patient perspectives [75]. The first meeting took place on 27 May 1995, in Berlin (Germany), with EGAN and the ESHG. WAO’s aims were formulated as follows: “Promote respect for persons with disabilities; promote protection from stigmatization of and discrimination against those affected with ’birth defects’, as well as those at risk for transmission of ‘birth defects’ to their children; increase dissemination of knowledge about prevention to parents affected” [76].

In 1997, the European Organization for Rare Diseases (EURORDIS), a non-profit alliance of RD/RGD patient organizations, was founded [77]. Under the inspiring leadership of the then-managing director, Yann le Cam, EURORDIS effectively assumed a key role in advocating for policies and policy initiatives at EU and international level. At the EU level, EURORDIS is driving and shaping EU policies on behalf of its members with the goal of influencing EU and national policymakers. To influence relevant legislation and policies, EURORDIS lobbies institutions governing the EU (i.e., the European Commission, the European Parliament, and the Council of the EU) to ensure that the needs and perspectives of individuals living with rare diseases are represented in decision-making processes.

EURORDIS played a significant role in the development of the Regulation of 1999 on Orphan Medicinal Products [78] and the subsequent Commission Communication on “Rare Diseases: Europe’s challenges” in 2008 [79], the Council Recommendation on an action in the field of rare diseases in 2009 [19], and the Directive 2011/24/EU of the European Parliament and of the Council of 9 March 2011 on the application of patients’ rights in cross-border healthcare (2011) [80]. Subsequently, EURORDIS lobbied for the EU member states’ recommendation to introduce a national plan (one for each country) on RDs [81]. In 2017, after years of advocacy from the Solve-RD community [82] and EURORDIS, the European Commission launched the 24 European Reference Networks [ERN], with each network covering the EU region, i.e., all member states of the EU. In parallel, both EURORDIS and the RD community launched 24 European Patient Advocacy Groups (ePAGs), one per ERN, working in partnership with clinicians and researchers [83]. One year after the ERNs’ establishment, EURORDIS set forth 10 recommendations for ERNs’ deeper integration at national levels [84]. Most of them were accepted in 2019 by the ERN Board of Member States, linking national efforts with a common strategy at EU level [85]. 

These actions paved the way for JARDIN (2024–2027), the EU joint action for integrating European Reference Networks (ERNs) into national health systems across 29 countries, including all EU countries, Norway, and Ukraine. JARDIN’s overall aim is “to enhance care for rare disease patients at supranational level”, i.e., to ensure timely, equal access to highly specialized healthcare in the EU and participating non-EU countries [86,87].

EURORDIS was a partner in the Rare 2030 foresight study, a two-year study that ended in February 2021, initiated by the European Parliament and co-funded by the European Commission Pilot Project and Preparatory Actions Programme. The study gathered the input of a large group of patients, practitioners, and key opinion leaders and proposed policy recommendations to improve policies for people living with a rare disease in Europe [88].

Internationally, EURORDIS is a member of the International Rare Diseases Research Consortium (IRDiRC) [89]. IRDiRC, established in 2011, brings together researchers and organizations invested in rare disease research. Three IRDiRC Scientific Committees (Diagnostics, Interdisciplinary, and Therapies) and representation from three patient advocacy groups (two from the US [the National Organization for Rare Disorders (NORD) and Genetic Alliance] and one from Europe [EURORDIS]) advise the consortium assembly, which includes public research funders and private-sector members from pharma and biotech, totalling 42 member institutions [90].

In December 2021, Rare Diseases International (RDI), the NGO Committee for Rare Diseases, and EURORDIS succeeded in securing the adoption of the first-ever United Nation (UN) Resolution on “Addressing the Challenges of Persons Living with a Rare Disease and their Families” [91,92,93].

Today, EURORDIS is an alliance of 1000+ rare disease patient organizations from 74 countries that work together to improve the lives of “300+ million people” living with a rare disease globally [94]. VSOP is an active member of EURORDIS and represents EURORDIS in The Netherlands.

In August 2001, my successor at the VSOP, C. Oosterwijk, took over the duties of managing director. The following organizations were founded in close collaboration with me to further patient advocacy interests:

In 2012, the European Patients’ Academy on Therapeutic Innovation (EUPATI), resulted from the EU-funded PATIENTPARTNER project [95]. It was co-ordinated by VSOP in co-operation with Genetic Alliance UK and the European Forum for Good Clinical Practice (EFGCP). EUPATI is a multi-stakeholder public–private partnership originally launched by the IMI-EUPATI project (European Patients’ Academy on Therapeutic Innovation, 2012–2017) and hosted by the European Patients’ Forum (EPF) from 2017 to 2020 [96]. Today, EUPATI is established as an independent non-profit foundation in The Netherlands [97].

Because some rare congenital disorders originate from a combination of individual genetic susceptibility in combination with external factors, we founded the Preparing for Life (PfL) initiative in 2012 [98]. The aim was to promote pre-conception health education and care worldwide, and to advocate the provision of biomedical, behavioural, and social health interventions on behalf of women and couples before conception occurs. We were able to realize a conference with WHO delegates and representative stakeholders from all parts of the world at WHO headquarters in Geneva, resulting in the WHO report *Preconception Care to Reduce Maternal and Childhood Mortality and Morbidity* in 2013 [99]. The report describes preconception risk factors and packages of interventions in thirteen domains for women and couples before conception that can improve subsequent pregnancy and child health outcomes.

Recent VSOP actions include:

1. The development of educational material about next-generation sequencing (“Next generation sequencing in diagnostiek”) for the public, patients, and healthcare providers in collaboration with Erfocentrum [100];
2. Joining of the supervisory board of H2O (Health Outcomes Observatory), a European project financed by both the EU and the European Federation of Pharmaceutical Industries and Associations (EFPIA) [101]. EFPIA represents the biopharmaceutical industry operating in Europe [102]. This project is intended to provide insight into clinical data and patient-reported data for patients from different disease areas and healthcare providers via dashboards, for example, for joint decision-making [103];
3. Close involvement—through support to access to empirical data- in the document “Advice from the Health Council of The Netherlands” (Gezondheidsraad) to the government in November 2023 on preconception carrier screening. The Health Council recommends pilot research to determine whether preconception carrier screening could be responsibly offered to all prospective parents in The Netherlands to equip them with the information they need to enable them to make informed reproductive decisions [104];
4. Co-ordinating the input of patients and patient organizations into the Clinical Genetics Knowledge Agenda, which describes the top 10 knowledge gaps in clinical genetic care. The two overarching knowledge questions are: what barriers are in place for healthy family members who are at increased risk of a hereditary condition with treatment options to be referred for genetic counselling and possibly DNA testing, and how can the identified barriers be overcome? The agenda was published at the end of 2022 [105]. The Netherlands Association of Clinical Geneticists (VKGN) is currently exploring the possibilities for further research to address these knowledge gaps, including input from the patient perspective.
5. Participation in the decision-making process to add an NIPT (Non-Invasive Prenatal Test) in April 2023 as a standard option—if the pregnant woman wishes so—in the prenatal screening program of The Netherlands [106].

VSOP advises The Netherlands Organization for Health Research and Development (ZonMw programs) on the development of national policies for RD/RGD [107], and is a member of other health and health-research platforms such as the Forum Biotechnology and Genetics [108]. A detailed summary of past VSOP RD/RGD activities and achievements is included in the EU 2017 RD Action report [109].

In October 2023, the House of Representatives of The Netherlands unanimously adopted a motion calling on the Minister of Health to investigate how an organization such as VSOP can be financed based on the number of member organizations (about a hundred for VSOP, while current financing is maximized at 15).

## 4. Where We Are Today, Future Prospects, and Challenges

When (V)SN was founded in 1967 out of the necessity to break individual isolation, no one could have foreseen the vibrant multi-layer ecosystem of RD/RGD patient organization networks existing today, nor could we have imagined the tremendous increase, progress, and achievements of these networks. Today, patients and their support organizations play an active role in shaping health and research policies at national, EU, and international levels to ensure that their needs and experiences are addressed [52,54]. These achievements include redefining the relationship as an equal partnership between researchers, clinicians, and patient groups.

Equally, in 1967, the stunning scientific progress of genetics and genomics was unimaginable. The transformative effects of this progress have been profound. Because of massively parallel next-generation DNA sequencing, the diagnosis of genetic diseases has advanced rapidly. Clinical genomics now provides a new form of genetic testing, “unprecedented in scope and comprehensiveness”, that has produced impressive diagnostic yield [110]. It enables medical genetics to use molecular diagnostics to analyse the genes of individual patients at affordable cost and acceptable turnaround time for clinical use [111].

### 4.1. Challenges

#### 4.1.1. Availability of Treatment and Costs of Cell and Gene Therapies

However, as the number of identified distinct genetic diseases keeps rising, only 6% (appr. 500) of these have established therapies, known as orphan therapies [112]. This leaves ~ 94% of RDs with no approved treatment [113]. As much as 77.3–80.7% of the population burden of rare diseases is attributable to the 4.2% (*n* = 149) of diseases in the most common prevalence range (1–5 per 10,000) [8]. However, the majority of RD/RGD are “ultra-rare” or “hyper-rare” [114].

While the costs of sequencing a human genome have tumbled down to approximately US$ 525 (delivery: 1 day) in 2022 as compared to US$ 100 million in 2003 (13 years), leading to a million-fold reduction in costs [115], therapeutic development costs are dauntingly expensive and time consuming. As a result, cell and gene therapies are prohibitively high-cost [116,117,118]. It is estimated that the clinical-stage R&D investment required to bring a new cell or gene therapy to market is US$ 1943 M (95% CI US$ 1395 M, US$ 2490 M) [119]. There is little or no monetary incentive for a business to develop one unique drug for a small number of patients. RD/RGD therapy development is about as expensive as common disease therapy, but eligibility is restricted, raising the per capita cost of treatment for RD/RGD patients to astronomically high levels. In addition, the standards for traditional clinical trials are not applicable because the majority of RGDs are “ultra-rare” or “hyper-rare”.

The high costs of development and treatment in relation to the rarity of each disease remains a challenge and impacts availability, accessibility, and affordability of cell and gene therapies [120,121]. The observable pricing developments raise concerns about unaffordability not only for RGD patients and their families but for health insurance systems as well [122]. This creates ethical dilemmas in terms of fair and equitable access to therapies, especially in societies with no universal healthcare coverage. How to solve the challenge of current high cell and gene therapy pricing, and how to ensure for patients fair and equitable access to affordable cell and gene therapies, needs to be addressed [123].

#### 4.1.2. Diagnostic Odyssey and Child Mortality

Today, the odds that a physician has seen or heard of a particular set of RGD symptoms are still slim. Siloed health data are common in many healthcare systems. This structure can prevent medical professionals from accessing the information they need in time. On average, it still takes 4.7 years in the EU between the onset of first symptoms and a confirmed diagnosis [124]. However, identifying the problem is only the start. Just as obtaining a diagnosis is time consuming and difficult, so too is having a treatment (see above). RGDs disproportionally affect children. An estimated 50–75% of rare diseases begin in childhood, and the lack of curative treatment options is still a key driver of mortality among children [125,126,127].

#### 4.1.3. Lack of Diversity in Genomics Studies

Lack of diversity in genomics is a global challenge: as of June 2021, the vast majority (86%) of genomics studies have been conducted in individuals of European descent, which represents an increase from 81% in 2016. At the same time, the proportion of studies conducted in underrepresented populations has either stagnated or decreased; genetic studies including participants with multiple ancestries have increased but only very slightly, to 4.8% [128].

The European ancestry bias in genomic study results and the continued underrepresentation of populations of mixed ancestry or of people whose ancestry is not European constitutes a problem. Until geneticists are able to conduct genome-wide association studies on each major ancestral population across the world, they will continue to miss important information about disease biology. They will not know how many of the thousands of associations between variants and diseases, and between variants and responses to drugs, observed in populations of European ancestry replicate in other groups [129].

### 4.2. Limitations

Spanning a timeline of more than 60 years, this historical account necessarily remains cursory and selective as it is written from a personal (subjective) perspective. Due to the wealth of data that has been accumulated over 60+ years, this account is by no means exhaustive. In addition, the paper concentrates primarily on the historic development of patient organizations for RGDs in Western countries, particularly in the EU and Europe. That is to say, the development of patient advocacy groups in high-income countries with universal health coverage and a tradition of civil society engagement [130].

## 5. Epilogue and the Lessons I Learned

It is 2024 now, and my daughter, despite the original dire prognosis of a short survival time in the 1960s, has a paid management job, is leading an autonomous life as normal as possible, and enjoys it. She has no strength in the muscles of her legs and hardly any in those of her arms. She moves around in an electrical wheelchair with an electrical arm-movement support, drives a minivan with a joystick, organizes and manages her own helpers 24/7 for daily tasks, and has many friends and hobbies. She studied at a conservatory and became a certified music teacher. Once her admission to the conservatory was a fact, she decided to skip all physiotherapy “*because it appeared to be nothing compared to the training scheme of the conservatory*”. She started diving and got a diving license during a holiday trip.

Looking back at my life, I realize that my daughter has been my guide in many ways. With respect to my personal life, she taught me the importance of the intrinsic gift of joy in life, of perseverance and social skills. I admire her because she manages to tackle all the difficulties that our society, which is designed for the average healthy person without limitations, puts in her way. She never complains but points out to competent authorities how they can remove obstacles to the normal functioning of people with disabilities. She never gets discouraged. With respect to my working life, she has inspired me at every step along the way I took on the road to improving knowledge about and diagnosis, care, and treatment of RGDs. Without this inspiration and motivation, I would never have been able to fulfil all the tasks I had taken on at the same time. And she still is my inspiration today when I give advice to the people I work with when they ask for it.

## Data Availability

The original contributions presented in the study are included in the article.
